# Estimated stroke risk, yield, and number needed to screen for atrial fibrillation detected through single time screening: a multicountry patient-level meta-analysis of 141,220 screened individuals

**DOI:** 10.1371/journal.pmed.1002903

**Published:** 2019-09-25

**Authors:** Nicole Lowres, Jake Olivier, Tze-Fan Chao, Shih-Ann Chen, Yi Chen, Axel Diederichsen, David A. Fitzmaurice, Juan Jose Gomez-Doblas, Joseph Harbison, Jeff S. Healey, F. D. Richard Hobbs, Femke Kaasenbrood, William Keen, Vivian W. Lee, Jes S. Lindholt, Gregory Y. H. Lip, Georges H. Mairesse, Jonathan Mant, Julie W. Martin, Enrique Martín-Rioboó, David D. McManus, Javier Muñiz, Thomas Münzel, Juliet Nakamya, Lis Neubeck, Jessica J. Orchard, Luis Ángel Pérula de Torres, Marco Proietti, F. Russell Quinn, Andrea K. Roalfe, Roopinder K. Sandhu, Renate B. Schnabel, Breda Smyth, Apurv Soni, Robert Tieleman, Jiguang Wang, Philipp S. Wild, Bryan P. Yan, Ben Freedman

**Affiliations:** 1 Heart Research Institute, Charles Perkins Centre, University of Sydney, Camperdown, New South Wales, Australia; 2 School of Mathematics and Statistics, University of New South Wales, Sydney, Australia; 3 Division of Cardiology, Department of Medicine, Taipei Veterans General Hospital, Taipei, Taiwan; 4 Institute of Clinical Medicine and Cardiovascular Research Center, National Yang-Ming University, Taipei, Taiwan; 5 The Shanghai Institute of Hypertension, Ruijin Hospital, Shanghai, China; 6 Shanghai Jiaotong University School of Medicine, Shanghai, China; 7 Department of Cardiology and Centre of Individualized Medicine of Arterial Disease, Odense University Hospital, Odense, Denmark; 8 Warwick Medical School, University of Warwick, Coventry, United Kingdom; 9 Servicio de Cardiologia, Hospital Universitario Virgen de la Victoria, Malaga, Spain; 10 CIBERCV, Malaga, Spain; 11 Discipline of Medical Gerontology, Trinity College Dublin, Dublin, Ireland; 12 The Irish Longitudinal Study of Ageing, Dublin, Ireland; 13 Population Health Research Institute, McMaster University, Hamilton, Ontario, Canada; 14 Nuffield Department of Primary Care Health Sciences, University of Oxford, Oxford, United Kingdom; 15 Julius Center for Health Sciences and Primary Care, University Medical Center Utrecht, Utrecht, the Netherlands; 16 Kaiser Permanente San Diego, San Diego, United States of America; 17 School of Pharmacy, Faculty of Medicine, The Chinese University of Hong Kong, Hong Kong; 18 Department of Vascular Surgery, Odense University Hospital, Odense, Denmark; 19 Liverpool Centre for Cardiovascular Science, University of Liverpool and Liverpool Heart and Chest Hospital, Liverpool, United Kingdom; 20 Aalborg Thrombosis Research Unit, Department of Clinical Medicine, Aalborg University, Aalborg, Denmark; 21 Department of Cardiology, Cliniques du Sud Luxembourg, Vivalia, Arlon, Belgium; 22 Primary Care Unit, Department of Public Health and Primary Care, University of Cambridge, Cambridge, United Kingdom; 23 University of Córdoba, Reina Sofia University Hospital, Unit of Family and Community Medicine of Córdoba, UGC Poniente, Córdoba and Guadalquivir Sanitary District, Córdoba, Spain; 24 Instituto Maimónides de Investigación Biomédica de Córdoba (IMIBIC), Córdoba, Spain; 25 Division of Cardiology, Department of Medicine, University of Massachusetts Medical School, Worcester, United States of America; 26 UMass Memorial Medical Center, Worcester, United States of America; 27 Universidade da Coruña, A Coruña, Spain; 28 Instituto Universitario de Ciencias de la Salud e Instituto de Investigación Biomédica de A Coruña, CIBERCV, A Coruña, Spain; 29 Center of Cardiology I, University Medical Center of the Johannes Gutenberg-University Mainz, Mainz, Germany; 30 Center for Translational Vascular Biology (CTVB), University Medical Center of the Johannes Gutenberg-University Mainz, Mainz, Germany; 31 DZHK (German Center for Cardiovascular Research), partner site RhineMain, Mainz, Germany; 32 School of Health and Social Care, Edinburgh Napier University, Edinburgh, Scotland; 33 Teaching Unit of Family and Community Medicine of Córdoba, Córdoba and Guadalquivir Sanitary District. Instituto Maimónides de Investigación Biomédica de Córdoba (IMIBIC), Córdoba, Spain; 34 Reina Sofía University Hospital, University of Córdoba, Córdoba, Spain; 35 Department of Clinical Sciences and Community Health, University of Milan, Milan, Italy; 36 Geriatric Unit, Fondazione IRCCS Ca’ Granda Ospedale Maggiore Policlinico, Milan, Italy; 37 Libin Cardiovascular Institute of Alberta, University of Calgary, Calgary, Alberta, Canada; 38 Cardiac Electrophysiology, Division of Cardiology, University of Alberta, Edmonton, Alberta, Canada; 39 University Heart Center Hamburg, Hamburg, Germany; 40 DZHK (German Center for Cardiovascular Research), Partner Site Hamburg/Kiel/Luebeck, Hamburg, Germany; 41 Department of Public Health Medicine, HSE West, Galway, Ireland; 42 Clinical and Population Health Research, Department of Quantitative Health Sciences, University of Massachusetts Medical School, Worcester, United States of America; 43 Department of Cardiology, Martini Hospital Groningen, Groningen, the Netherlands; 44 Department of Cardiology, University Medical Center Groningen, Groningen, the Netherlands; 45 Preventive Cardiology and Preventive Medicine, University Medical Center of the Johannes Gutenberg-University Mainz, Mainz, Germany; 46 Center for Cardiology, University Medical Center of the Johannes Gutenberg-University Mainz, Mainz, Germany; 47 Center for Translational Vascular Biology (CTVB), University Medical Center of the Johannes Gutenberg-University Mainz, Mainz, Germany; 48 Center for Thrombosis and Hemostasis, University Medical Center of the Johannes Gutenberg-University Mainz, Mainz, Germany; 49 Division of Cardiology, Department of Medicine and Therapeutics, The Chinese University of Hong Kong, Hong Kong SAR, China; 50 Prince of Wales Hospital, Hong Kong SAR, China; Columbia University, UNITED STATES

## Abstract

**Background:**

The precise age distribution and calculated stroke risk of screen-detected atrial fibrillation (AF) is not known. Therefore, it is not possible to determine the number needed to screen (NNS) to identify one treatable new AF case (NNS-Rx) (i.e., Class-1 oral anticoagulation [OAC] treatment recommendation) in each age stratum. If the NNS-Rx is known for each age stratum, precise cost-effectiveness and sensitivity simulations can be performed based on the age distribution of the population/region to be screened. Such calculations are required by national authorities and organisations responsible for health system budgets to determine the best age cutoffs for screening programs and decide whether programs of screening should be funded. Therefore, we aimed to determine the exact yield and calculated stroke-risk profile of screen-detected AF and NNS-Rx in 5-year age strata.

**Methods and findings:**

A systematic review of Medline, Pubmed, and Embase was performed (January 2007 to February 2018), and AF-SCREEN international collaboration members were contacted to identify additional studies. Twenty-four eligible studies were identified that performed a single time point screen for AF in a general ambulant population, including people ≥65 years. Authors from eligible studies were invited to collaborate and share patient-level data. Statistical analysis was performed using random effects logistic regression for AF detection rate, and Poisson regression modelling for CHA_2_DS_2_-VASc scores. Nineteen studies (14 countries from a mix of low- to middle- and high-income countries) collaborated, with 141,220 participants screened and 1,539 new AF cases. Pooled yield of screening was greater in males across all age strata. The age/sex-adjusted detection rate for screen-detected AF in ≥65-year-olds was 1.44% (95% CI, 1.13%–1.82%) and 0.41% (95% CI, 0.31%–0.53%) for <65-year-olds. New AF detection rate increased progressively with age from 0.34% (<60 years) to 2.73% (≥85 years). Neither the choice of screening methodology or device, the geographical region, nor the screening setting influenced the detection rate of AF. Mean CHA_2_DS_2_-VASc scores (*n* = 1,369) increased with age from 1.1 (<60 years) to 3.9 (≥85 years); 72% of ≥65 years had ≥1 additional stroke risk factor other than age/sex. All new AF ≥75 years and 66% between 65 and 74 years had a Class-1 OAC recommendation. The NNS-Rx is 83 for ≥65 years, 926 for 60–64 years; and 1,089 for <60 years. The main limitation of this study is there are insufficient data on sociodemographic variables of the populations and possible ascertainment biases to explain the variance in the samples.

**Conclusions:**

People with screen-detected AF are at elevated calculated stroke risk: above age 65, the majority have a Class-1 OAC recommendation for stroke prevention, and >70% have ≥1 additional stroke risk factor other than age/sex. Our data, based on the largest number of screen-detected AF collected to date, show the precise relationship between yield and estimated stroke risk profile with age, and strong dependence for NNS-RX on the age distribution of the population to be screened: essential information for precise cost-effectiveness calculations.

## Abstract 2

Nicole Lowres and colleagues report on stroke risk in people with atrial fibrillation detected by screening.

## Author summary

**Why was this study done?:**

- Atrial fibrillation is a common heart rhythm problem that often has no symptoms, so people are unaware they have this condition.
- People with atrial fibrillation can have a very high stroke risk if they are not appropriately treated with anticoagulant medications, and this risk increases with age.
- Screening for atrial fibrillation is recommended in many guidelines, although the precise age distribution and calculated stroke risk of atrial fibrillation detected by screening is not known.
- Accurate age-specific data are required for cost-effectiveness analysis, to inform the most appropriate age cutoff for screening based on the age distribution of the population to be screened.

**What did the researchers do and find?:**

- Investigators from 19 atrial fibrillation screening studies across the world agreed to collaborate and share patient-level data, providing a combined database of 141,220 people screened and 1,539 screen-detected cases of atrial fibrillation.
- Our study was able to quantify the yield and stroke risk for atrial fibrillation in 5-year age brackets, showing the exact relationship of how the yield of screening and stroke risk of screen-detected atrial fibrillation increases with age.
- The yield of screening was not influenced by the screening method used or the recruitment setting, indicating that screening programs can be established based on available resources.
- To our knowledge, this is the first study to demonstrate the precise relationship of the number that need to be screened to identify one new atrial fibrillation case, or one new atrial fibrillation case in whom anticoagulant treatment is guideline recommended, in 5-year age brackets.

**What do these findings mean?:**

- This study demonstrates the high calculated stroke risk of screen-detected AF and the high proportion with at least one additional stroke risk factor other than age or sex.
- These data allow for accurate simulations of cost-effectiveness of screening, including sensitivity analyses, based on the age distribution of the population to be screened.
- Ultimately, these data may be used to assist development of health policy around the development of atrial fibrillation screening programs, tailored to the specific health system and resources available.

## Introduction

The role of opportunistic or systematic atrial fibrillation (AF) screening for people aged ≥65 years remains contested, with variation in recommendations between international AF clinical guidelines. However, 10% of all ischaemic strokes are in individuals with undiagnosed AF [1], and early identification of AF and appropriate guideline-based oral anticoagulation (OAC) treatment can prevent strokes and thus reduce health costs related to AF [2]. Organisations supporting the recommendation to screen include the European Society of Cardiology (ESC) [3], the European Heart Rhythm Association [4], the Royal College of Physicians of Edinburgh [5], AF-SCREEN International Collaboration [6], and, recently, the Heart Foundation of Australia and the Cardiac Society of Australia and New Zealand [7].

The evidence to support screening has mainly been extrapolated from studies of people with clinically or incidentally diagnosed AF and from prevalence studies that show both AF prevalence and stroke risk increase substantially from age 65. No large outcome trial of screen-detected AF using hard events, including stroke and death, has been reported to date. Few studies have reported the baseline estimated stroke risk of screen-detected AF patients. In the screening for atrial fibrillation in the elderly (SAFE) trial, the calculated stroke risk was the same in screen-detected and symptomatically identified AF patients [8], but it was not possible to accurately determine the stroke risk in discrete age strata or the number needed to screen (NNS) to identify one treatable new AF case (NNS-Rx) in each age stratum. This information is important for precise cost-effectiveness and sensitivity simulations based on the age distribution of the population to be screened. Such calculations are required by payers to determine the best age cutoffs for screening programs and decide whether programs of screening should be funded.

We therefore performed a systematic review and meta-analysis to investigate the yield of new AF identified in contemporary AF screening studies (single time point) and to explore the stroke risk profile and OAC eligibility of those identified, in order to determine the precise age distribution and calculated stroke risk of AF in 5-year age strata to enable accurate cost-effectiveness modelling.

## Methods

This systematic review and patient-level meta-analysis was performed in accordance with the preferred reporting items for systematic reviews and meta-analysis (S1 PRISMA checklist) and the meta-analyses of observational studies in epidemiology guidelines [9,10]. All collaborating studies had ethical approval for their study, the details of which are reported in the individual study manuscripts [11–29]. Ethical approval was not required for this collaborative secondary analysis of data.

### Search strategy and selection criteria

Relevant studies were identified by two independent reviewers (NL and BF) through electronic database searching of MEDLINE, Pubmed, Embase, and Google. The keyword search terms used were as follows: atrial fibrillation AND [screening OR incidence OR prevalence OR detection OR identification] up to February 2018. To ensure a relevant contemporary sample was obtained, limits were applied to years 2007 onwards, and human research. Studies published in any language were permitted. Additional studies were identified through directly contacting members of the AF-SCREEN International Collaboration [6]. Study authors from all eligible studies were contacted via email, with an explanation of the proposed study and an invitation to collaborate.

The inclusion criteria for screening studies were as follows: (i) evaluated a general ambulant population; (ii) included people ≥65 years within their screened population; (iii) used a valid method to identify AF, as accepted by the ESC 2016 AF guidelines (i.e., pulse palpation, 12-lead electrocardiogram [ECG], or ECG rhythm strip, with a validated device) [3]; (iv) assessed the rate of newly identified AF using a single time point screen; (v) distinguished between newly identified AF and previously diagnosed AF; (vi) screened a sample size of at least 1,000 people; (vii) collected participant age and gender for all new AF; and (viii) collected participant age for all participants screened. Studies were excluded if they performed repeated, intermittent, or continuous recordings over a period to identify unknown AF or if screening was targeted at a specific subgroup (e.g., limited age range, hypertension, diabetes, poststroke).

Assessment of Quality of Reporting was not performed, as some participating studies had not published their results. However, to ensure only studies of appropriate quality were included, our study inclusion criteria were intentionally developed based on the modified Newcastle-Ottawa scale criteria, specifically (i) the source population is representative, (ii) past history of AF is ascertained, (iii) a validated measurement tool is used, (iv) sample size is adequate, (v) methodology is appropriate for outcomes, and (vi) variables are clearly defined.

### Study outcomes

The primary study outcome was the detection rate for cases of new AF identified through screening of people aged ≥65 years with one screen at a single time point (reported as [number of positive cases/100 persons screened] and 95% CI). Secondary outcomes of interest were (i) detection rate for cases of new AF identified through screening with one screen at a single time point, stratified according to each age group (<60, 60–64, 65–69, 70–74, 75–79, 80–84, and ≥85 years) (reported as [number of positive cases/100 persons screened] and 95% CI); (ii) CHA_2_DS_2_-VASc stroke risk score, stratified according to age group (reported as means and 95% CI); (iii) eligibility for OAC according to ESC 2016 guidelines, stratified according to age group (reported as number and percentage); (iv) proportion of new AF cases with stroke risk factors other than age and sex (i.e., chronic heart failure, hypertension, diabetes, prior stroke or transient ischaemic attack, or vascular disease), stratified according to age group (reported as number and percentage); (v) NNS to identify 1 new AF case for age ≥65 years, stratified according to age group; and (vi) NNS-Rx (i.e., new AF with a Class-1 recommendation to prescribe OAC) for age ≥65 years, stratified according to age group.

### Statistical analysis

Data from each study were exported into Microsoft Excel (version 1802) and checked for errors. Data fields collected from each study are summarised in S1 Text. Descriptive analyses were carried out to describe characteristics of participating studies, total numbers screened, and total numbers of AF identified through screening, stratified according to age group and sex.

#### Detection rate of new AF

The number of new AF cases among those screened was assumed to follow a binomial distribution, as only a binary outcome was possible from screening each participant (AF positive or AF negative). In accordance with our statistical analysis plan, the detection rate of new AF cases was estimated by random effects logistic regression. As binary data are unlikely to have a ‘normal distribution’, random effects logistic regression is preferred over conventional meta-analysis approaches that assume study-level effect sizes are normally distributed [30]. The consequence of choosing this approach is that the standard meta-analytic methods for detecting heterogeneity and publication bias cannot be applied. Heterogeneity was therefore assessed using the study-level random effect and standard error.

Individual-level data were available for the screening outcome (AF positive or AF negative), sex, and age group. Study-level information was available for country, geographical region, urban/rural population, screening method/device, screening setting/design, era screened, and screening age eligibility. Due to the combination of both individual and study-level data, the individual-level data were modelled first, and then the study-level variables were added. Study was included as a random effect in all models.

For the individual-level data, three models were considered: the intercept only (overall mean), then the addition of age groups, and then gender. The appropriateness of including each variable was based on comparison of the Akaike information criterion for each model. The study-level covariates were then added to the model one at a time, and the Akaike information criterion was used to determine if they should be included or not, based on comparison to the Akaike information criterion of the final individual-level model.

Individual logistic regression models were used for study-level estimates, and summary estimates were computed from a random effects logistic regression model using SAS GLIMMIX (v9.4) while adjusted for covariates. Age group estimates were computed using least square means from the final random effects logistic regression model. The results of the analysis from SAS GLIMMIX were imported into R, and the metafor package (R 3.4.3 ‘Kite-Eating Tree’) was used to create a forest plot. The results were reported for the age group ≥65 years and also stratified according to each age group (<60, 60–64, 65–69, 70–74, 75–79, 80–84, ≥85 years).

#### Stroke risk profile of new AF cases

Stroke risk of new AF cases was determined using the CHA_2_DS_2_-VASc score (range 0–9 points), which is the sum of risk factors: congestive heart failure/left ventricular dysfunction (1 point); high blood pressure (1 point); age >75 years (2 points); diabetes (1 point); stroke/transient ischaemic attack/thromboembolism (2 points); vascular disease (coronary artery disease, myocardial infarction, peripheral artery disease, aortic plaque) (1 point); age 65–74 years (1 point); and sex category female (1 point). The CHA_2_DS_2_-VASc score was chosen to measure stroke risk as it is recommended by most international guidelines [3,7,31], and it has demonstrated accuracy identifying AF patients who are at low risk of stroke and therefore do not require OAC [32,33].

Random effects Poisson regression modelling was performed for the CHA_2_DS_2_-VASc score. As the maximum data value of the CHA_2_DS_2_-VASc score is 9, we modelled the Poisson mean for the data (1.04) and calculated the probability that the value could be larger than 9 (1.58 × 10^−7^) to ensure that truncation of data relative to the Poisson distribution was not an issue.

For Poisson regression with study as a random effect, age groups were included to stratify the CHA_2_DS_2_-VASc mean estimates according to age brackets. The study-level covariates (i.e., geographical region, country, rural/urban population) were then added to the model one at a time, and the Akaike information criterion was used to determine if they should be included or not, based on comparison to the Akaike information criterion of the individual-level model. The mean CHA_2_DS_2_-VASc scores were similar for each country, with one exception. The mean score for this country was 1.7 (CI 1.2–2.4), while the next lowest was 2.4 (CI 1.6–3.5). The inclusion of this country could unduly influence the overall summary estimates. To assess the impact of these data in a sensitivity analysis, the model was refit without data from this country, and the summary estimates were compared. The final model included data from all countries.

Guideline recommendations for OAC were calculated for each new AF case with CHA_2_DS_2_-VASc score and sex data. The ESC 2016 guidelines were used to classify OAC recommendations into (i) Class-1 OAC recommendation (CHA_2_DS_2_-VASc score: men ≥ 2; women ≥ 3), (ii) consider OAC (CHA_2_DS_2_-VASc score: men = 1; women = 2), or (iii) OAC not recommended (CHA_2_DS_2_-VASc score: men = 0; women = 1) [3]. Data are reported as pooled number and percentages for each category and stratified according to age group (<60, 60–64, 65–69, 70–74, 75–79, 80–84, ≥85 years).

The number of additional stroke risk factors other than age and sex were calculated for each person with new AF using the formula, CHA_2_DS_2_-VASc score − female sex point − age points, and reported as a pooled percentage of all new AF, stratified according to age group.

#### NNS

The NNS to identify one new AF case was calculated using the inverse of the detection rate derived from the meta-regression, stratified according to age group. The NNS-Rx was calculated using the inverse of the determined yield of newly identified AF with a 2016 ESC Class-1 recommendation for OAC, stratified according to age group.

## Results

**Fig 1:**
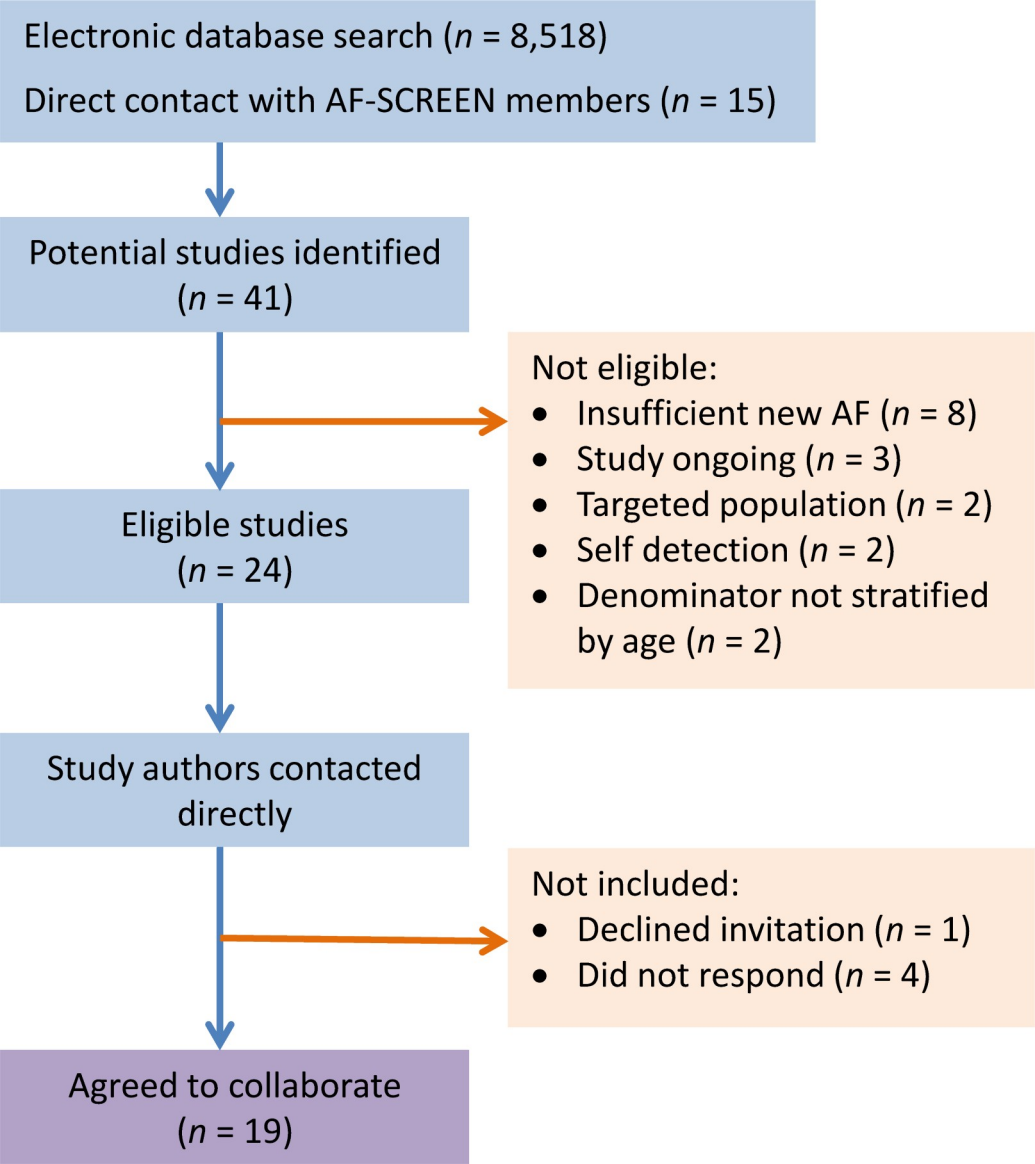
Study selection.

The search strategy identified 41 screening studies, of which 17 did not meet the eligibility criteria (Fig 1). Study authors from the 24 eligible studies were contacted via email, and 19 studies [11–29] from 14 countries agreed to the collaboration and contributed screening data.

A combined total of 141,220 participants were screened (approximately 44% men; sample size range, 1,000–59,505) (Table 1). Rates of detection of AF ranged from 0.35% in studies recruiting ≥40 years to 2.34% in studies recruiting ≥65 years. Studies recruited from community or population screening (*n* = 7), general practice (*n* = 6), outpatient clinics (*n* = 3), and pharmacies (*n* = 3). The screening methods used were single-lead ECG (*n* = 12), 12-lead ECG (*n* = 4), pulse palpation (*n* = 2), and modified blood pressure machine (*n* = 1).

**Table 1:** Characteristics of studies.

Author, year	Country, study name	Setting	Screening method	Year screened	Age Eligibility (years)	Number screened
Proietti and colleagues, 2016 [16]	Belgium, Belgian Heart Rhythm Week	Community/population	Single-lead ECG (Omron HCG-801)	2010–2014	≥20	59,505
Schnabel and colleagues, 2012 [11]	Germany, Gutenberg Health Study	Community/population	12-lead ECG	2007–2017	35–74	14,937
Yan and colleagues, 2017 [12]	Hong Kong	Outpatient clinic	Single-lead ECG (AliveCor)	2015–2017	≥40	12,928
Gomez-Doblas and colleagues, 2014 [13]	Spain, OFRECE	Community/population	12-lead ECG	2010–2012	≥40	8,396
Deif and colleagues, 2013 [14]	Australia	Outpatient clinic	12-lead ECG	2011	≥40	3,430
Soni and colleagues, 2017 [15]	India	Community/population	Single-lead ECG (AliveCor)	2016–2017	≥50	1,947
Li and colleagues, 2015 [17]	China	Community/population	12-lead ECG	2006–2011	≥60	3,922
Smyth and colleagues, 2016 [18]	Ireland	General practice	Pulse palpation (confirmed with 12-lead ECG)	2014	≥60	7,262
Chao and colleagues, 2017 [19]	Taiwan, SAFE-Taiwan	Pharmacy	Modified blood pressure device (Microlife WatchBP Office AFIB)	2015–2016	≥60	2,672
Kvist and colleagues, 2017 [20]	Denmark, DANCAVAS	Community/population	Single-lead ECG (Lead-II during Cardiac-CT scan)	2015–2016	65–74	1,318
Kaasenbrood and colleagues, 2016 [21]	the Netherlands	General practice	Single-lead ECG (MyDiagnostick)	2013	≥65	2,557
Lowres and colleagues, 2014 [22]	Australia, SEARCH-AF	Pharmacy	Single-lead ECG (AliveCor)	2012–2013	≥65	1,000
Sandhu and colleagues, 2016 [23]	Canada, PIAAF-Pharmacy	Pharmacy	Single-lead ECG (HeartCheck, CardioComm)	2014–2015	≥65	1,145
Quinn and colleagues, 2018 [24]	Canada, PIAAF-Family Practice	General practice	Single-lead ECG (HeartCheck, CardioComm); modified blood pressure device (Microlife WatchBP Home A); and pulse palpation (confirmed with 12-lead ECG ± holter)	2016–2017	≥65	2,054
González Blanco and colleagues, 2017 [25]	Spain, DOFA	General practice	Pulse palpation (confirmed with 12-lead ECG)	2015–2016	≥65	7,063
Fitzmaurice and colleagues, 2007 [26]	England, SAFE (systematic screening arm)	General practice	12-lead ECG	2001–2003	≥65	2,357
Orchard and colleagues, 2018 [27]	Australia, AF-SMART	General practice	Single-lead ECG (AliveCor)	2016–2017	≥65	1,574
Keen and colleagues, 2017 [28]	United States	Outpatient clinic	Single-lead ECG (AliveCor)	2016–2017	≥65	2,732
Wang and colleagues, 2017 [29]	China	Community/population	Single-lead ECG (AliveCor)	2017–2018	≥65	4,421

Abbreviations: AF-SMART, atrial fibrillation screen management and guideline recommended therapy; DANCAVAS, Danish Cardiovascular Screening trial; DOFA, Detección Oportunista de Fibrilación Auricular en Atención Primaria Study; ECG, electrocardiogram; OFRECE, Observación de FibRilacion auricular y Enfermedad Coronaria en España; PIAAF-Pharmacy, Program for the identification of ‘actionable’ atrial fibrillation in the pharmacy setting; PIAAF-Family Practice, Program for the identification of ‘actionable’ atrial fibrillation in family practice; SAFE, screening for atrial fibrillation in the elderly; SAFE-Taiwan, screen of atrial fibrillation events in Taiwan; SEARCH-AF, Screening education and recognition in community pharmacies of atrial fibrillation.

### New AF cases

**Fig 2:**
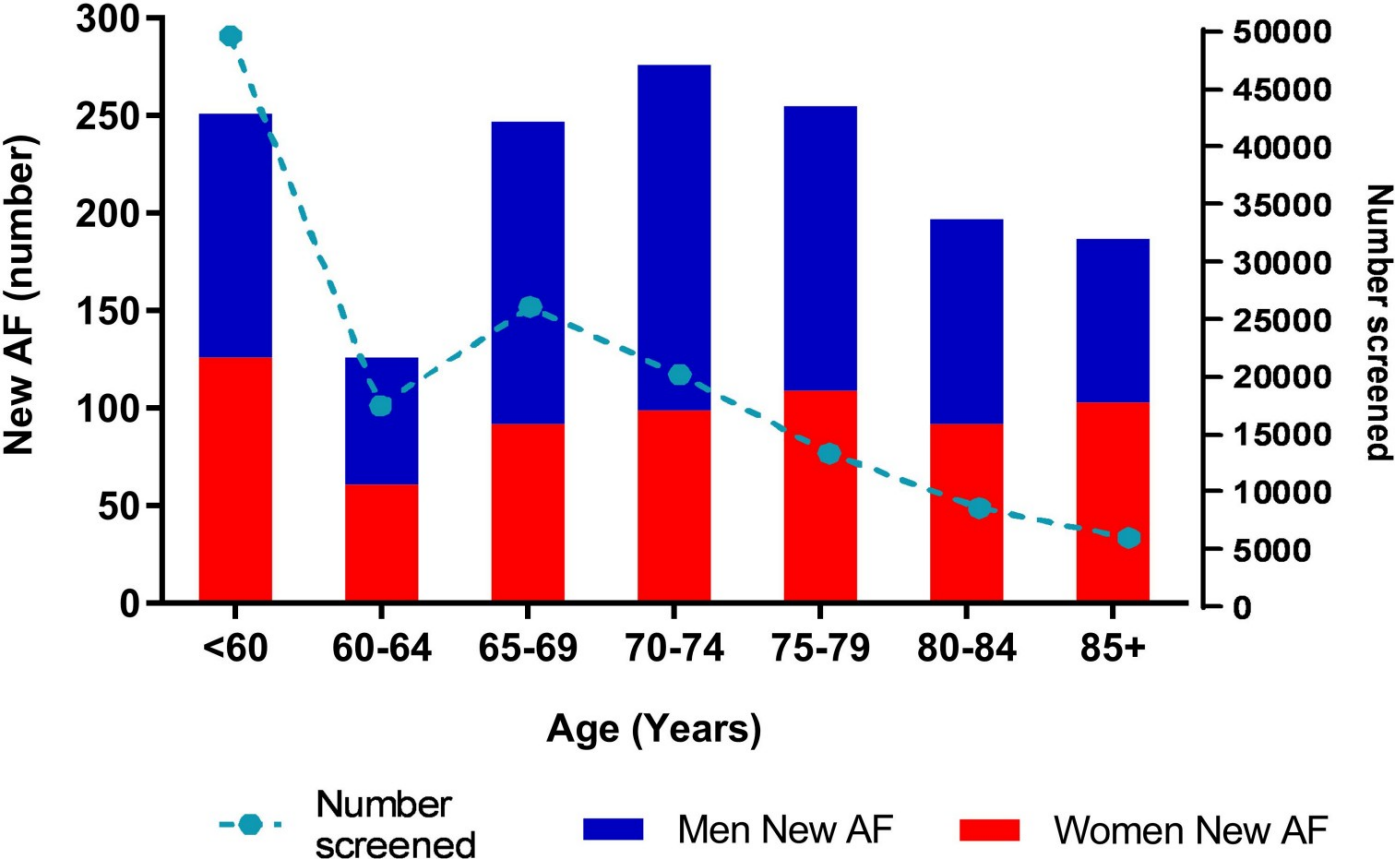
Total numbers of new AF by sex. AF, atrial fibrillation.

**Fig 3:**
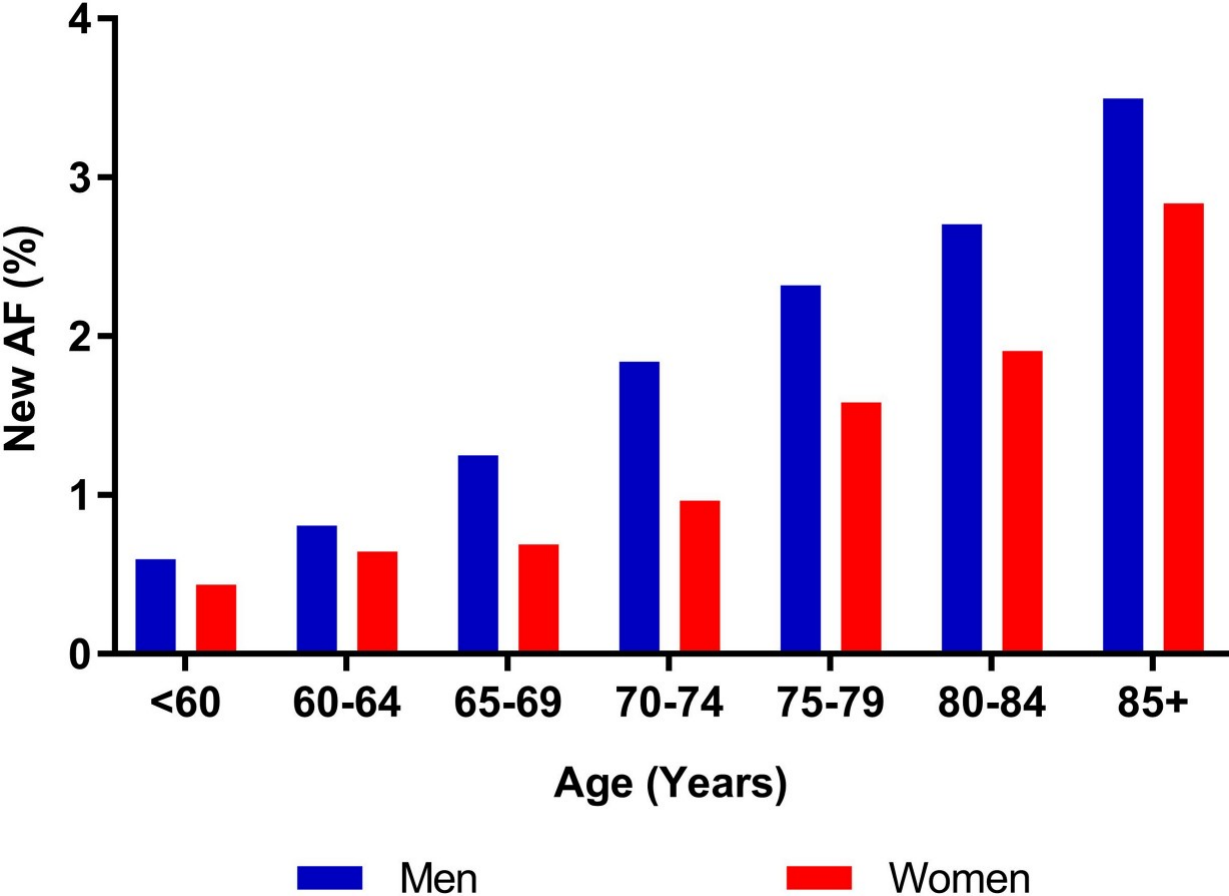
AF pooled yield by sex. AF, atrial fibrillation.

From the pooled data (*n* = 19 studies), 1,539 new cases of AF were identified from 141,220 participants screened. Limiting the results to people ≥65 years, 1,162 new cases of AF were identified from 74,104 participants screened. Absolute numbers of new AF identified were greatest within the range of 70–74 years (Fig 2). The pooled yield of screening was greater in males across all age strata and increased in both men and women with increasing age (Fig 3).

### AF detection rate

The inclusion of sex, age group, and cohort improved the fit of the random effects logistic regression model. The variables of setting, method, region, country, urban/rural, era screened, and screen age eligibility did not appear to influence the results. The final model was adjusted for age group and sex, and incorporated 18/19 studies (*n* = 138,663) for which data on total numbers screened were stratified by both age and sex [11–20,22–29]. The study-level random effect estimate was 0.2320 (SE = 0.0889), indicating a heterogeneous sample.

**Fig 4:**
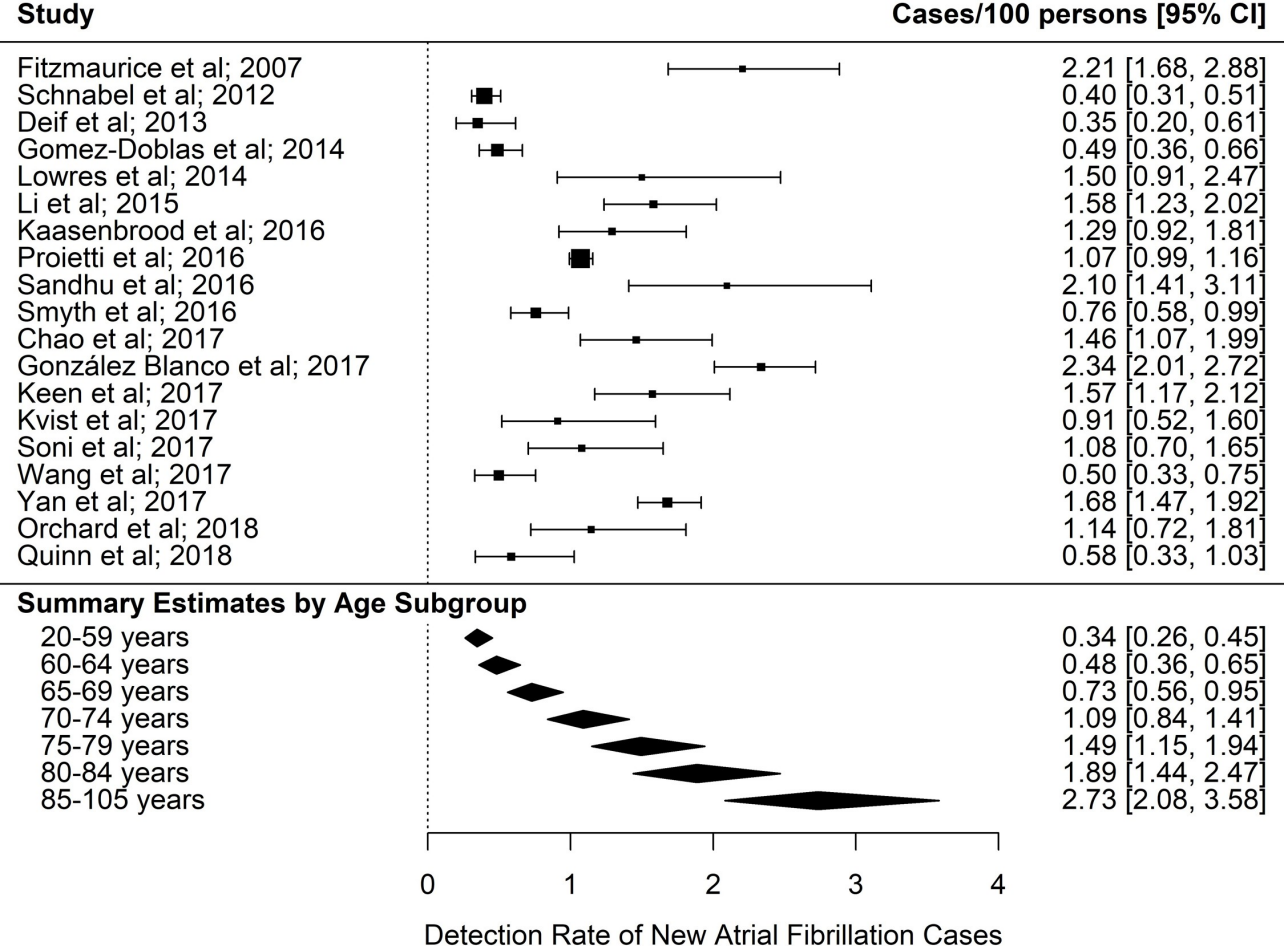
AF detection rate (adjusted for age and sex). Summary estimates are calculated from the 18/19 studies that provided both gender and age for total numbers screened. AF, atrial fibrillation.

**Fig 5:**
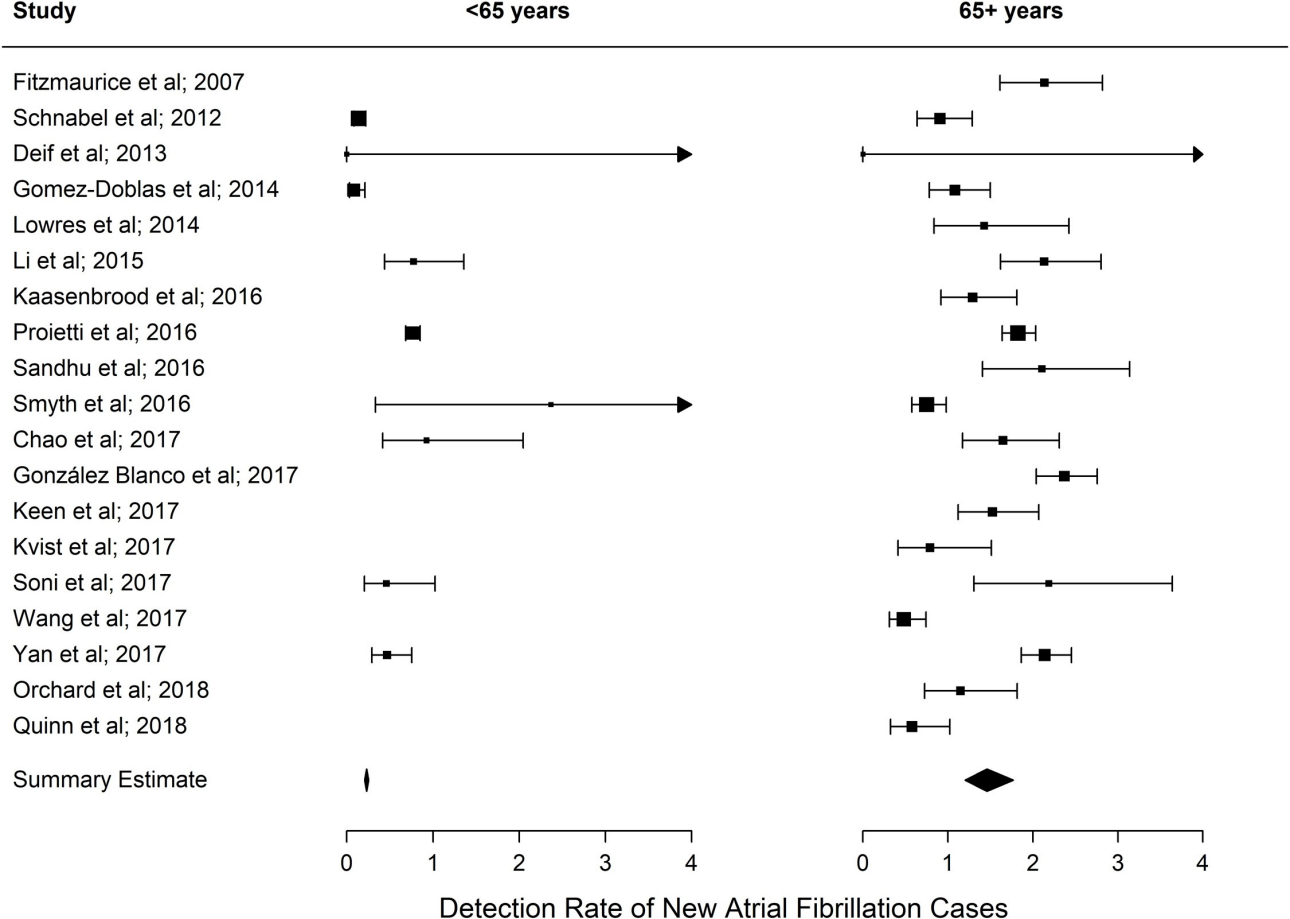
AF detection rate for <65 years and 65+ years. Summary estimates are calculated from the 18/19 studies that provided both gender and age for total numbers screened. AF, atrial fibrillation.

The detection rate for cases of new AF identified through screening increased progressively with increasing age, as presented in the summary estimates (Fig 4). Below age 60 years yield was 0.34%, increasing to 2.73% for ages 85 years and over. For screening people ≥65 years (as per guideline recommendations), the detection rate of new AF was 1.44% (95% CI, 1.13%–1.82%), compared with only 0.41% (95% CI, 0.31%–0.53%) for people aged <65 years (rate ratio = 3.57, 95% CI, 3.10–4.10) (Fig 5).

### Stroke risk profile

CHA_2_DS_2_-VASc scores were available for 1,369 new AF cases, collected at the time of screening, from 18/19 studies [11–24,26–29]. As expected, mean CHA_2_DS_2_-VASc scores increased progressively with age, with step increases at ages 65 and 75 years (Table 2). CHA_2_DS_2_-VASc results appeared to be influenced by a country/cohort effect, with the highest CHA_2_DS_2_-VASc means (>3.0) observed in Germany, Hong Kong, and America and the lowest (<2.0) in India. The results did not appear to be influenced by setting, method, urban/rural, era screened, or screen age eligibility.

**Table 2:** Stroke risk profile of new AF cases (*n* = 1,369).

Age group, years	Number, *n*	CHA_2_DS_2_-VASc, mean* (95% CI)	≥1 non-age/sex stroke risk factor, percent of age group	Guideline Recommendation^†^		
				No OAC, percent	Consider OAC, percent	Prescribe OAC, Class-1 percent
<60	251	1.1 (0.7–1.5)	46	54	19	27
60–64	125	1.4 (1.2–1.6)	54	45.5	32	22.5
65–69	223	2.5 (2.2–2.8)	65	0	35	65
70–74	240	2.7 (2.4–2.9)	69	0	32.5	67.5
75–79	228	3.8 (3.4–4.1)	76	0	0	100
80–84	151	3.8 (3.4–4.2)	75	0	0	100
85+	151	3.9 (3.6–4.4)	77	0	0	100

*Least square means.

^†^Recommendation according to the 2016 ESC AF guidelines.

Abbreviations: AF, atrial fibrillation; CHA_2_DS_2_-VASc score, (congestive heart failure/left ventricular dysfunction, high blood pressure, age >75 years, diabetes, stroke/transient ischaemic attack/thromboembolism, vascular disease [coronary artery disease, myocardial infarction, peripheral artery disease, aortic plaque], age 65–74 years, sex category female); ESC, European Society of Cardiology; OAC, oral-anticoagulation.

When considering only ‘non-age and non-sex’ factors of the CHA_2_DS_2_-VASc score, 72% (712/993) of new AF ≥65 years had at least one additional stroke risk factor (comorbidity) other than age or sex (Table 2). The number with comorbidities was lower in age groups 65–69 and 70–74 years (65% and 69%, respectively); however, it was >75% in all three age strata over 75 years.

Above age 65 years, the clear majority (84%) of screen-detected new AF was eligible for OAC with a Class-1 recommendation, according to the 2016 ESC guidelines (Table 2) [3]. For people aged ≥75 years, 100% had a Class-1 recommendation because of age alone. In the age range 65–74 years, 66% received a Class-1 recommendation, and the remaining 34% had a recommendation to consider OAC (Table 2). In contrast, for those <65 years, only 26% received a Class-1 recommendation, 23% had a recommendation to consider OAC, and half (51%) had a recommendation to not prescribe OAC (Table 2).

### NNS

When screening people ≥65 years, the NNS to identify one new AF is 69, rising to 83 to identify one treatable new AF (i.e., those with a Class-1 OAC recommendation). A progressive increase was observed in both NNS to identify one new AF and NNS-Rx as the age group decreased (Table 3). Specifically, there was a large jump noted between age 65–69 to 60–64 years, in which the NNS-Rx rose steeply from 211 to 926, and a further increase to 1,089 for people aged <60 years (Table 3).

**Table 3:** NNS.

Age group, years	NNS to identify 1 new AF (*n*)	NNS to identify 1 treatable new AF (*n*)^‡^
<60	294	1,089
60–64	208	926
65–69	137	211
70–74	92	136
75–79	67	67
80–84	53	53
85+	37	37

^‡^Newly identified AF with a Class-1 recommendation to prescribe OAC.

Abbreviations: AF, atrial fibrillation; NNS, number needed to screen; OAC, oral anticoagulation.

## Discussion

To our knowledge, this is the first study to show the actual yield of screen-detected AF and estimated stroke risk by age group, in very large numbers. Our data show that both yield and stroke risk are very sensitive to age, and the estimated stroke risk profile of new cases is high. When screening ≥65 years, the detection rate of new AF cases is 1.44% (95% CI, 1.13%–1.82%), and 84% of new AF cases have a Class-1 recommendation for OAC prophylaxis. Of note, under the 2016 Canadian AF Guidelines, all people aged ≥65 years receive an OAC recommendation based on age alone [34]. The high stroke risk profile is not solely due to age and sex, as 72% of new cases aged ≥65 years have at least one additional CHA_2_DS_2_-VASc stroke risk factor (comorbidity) other than age or sex. As expected, with increasing age there is a corresponding continuous increase in the detection rate of new AF, mean CHA_2_DS_2_-VASc scores, and additional CHA_2_DS_2_-VASc stroke risk factors. The yield of screening was higher in men across all age groups, even though larger numbers of women were screened.

The detection rate of 1.44% for screening people ≥65 years is comparable to the result of 1.4%, determined from a systematic review of AF screening in 2013 [35]. Both of these results are based on single time point screening and, as such, may be an underestimate of undetected AF, as some cases of paroxysmal AF may be missed. Intermittent or continuous screening over two weeks or longer will identify additional cases of paroxysmal AF, leading to a larger yield [36–38]. Indeed, only one sixth of new AF cases were detected at baseline ECG testing in the STROKESTOP trial, with the remainder detected during the subsequent two weeks of intermittent screening [36]. The REHEARSE-AF study detected 3.8% with new AF by 1–2 ECGs per week over 1 year, although in that study, 1.8% of patients screened for eligibility by a single ECG had new AF detected [39]. With two weeks of ambulatory ECG monitoring using an adhesive patch in the mSToPS study, 5.1% were detected with new AF [38]. Although additional new AF cases are identified and the cost-effectiveness of intermittent screening has been demonstrated in a targeted population of 75-year-olds, intensive screening is more expensive, and stroke risk is lower for the most intensive screening programs (e.g., implanted cardiac monitors) [40]. Therefore, intensive screening is not currently recommended for a generalised population [41]. For this reason, this review focused solely on single time point screening, as it corresponds with clinical practice and is well suited for opportunistic screening according to guideline recommendations.

For implementation of opportunistic screening, our review indicates that the choice of screening setting and the methodology/device chosen to screen (i.e., pulse palpation, single-lead ECG, 12-lead ECG, or modified blood pressure machine) do not influence the detection rate. Therefore, decisions on how to implement screening can be tailored to available local or national resources, practice preference, the requirements of the health system, and the population to be screened. Decisions around developing a screening program also critically require consideration of the pathway to treatment, as 84% of new AF identified (aged ≥65 years) will require a consultation for consideration of OAC prescription.

Our data do not support screening a general population younger than 65 years, as the yield is low, and only 26% of new AF cases would receive a Class-1 recommendation to treat with OAC. Even to consider screening people aged 60–64, the NNS-Rx increases markedly to 926, compared with 211 for ages 65 to 69 years. For the population below 60 years, to identify one treatable person requires screening 1,089 people. Screening people younger than 65 may be appropriate in targeted populations (e.g., poststroke or in those with additional stroke risk factors), as both yield and stroke risk profile are likely to be higher, in which case the NNS-Rx would reduce significantly [42,43].

The NNS data will be very important to determine precise estimates of cost-effectiveness. To date, health-economic analyses from many countries, based on a similar yield of new AF, have all demonstrated the likely cost-effectiveness of AF screening based on quality-adjusted life years gained and strokes avoided [8,22,41,44–46]. Cost-effectiveness is sensitive to OAC prescription rates and improves as OAC prescription rates increase [22]. Given the recent trend of increased guideline-based prescription rates from 48% to 78.6% noted in the United Kingdom since the introduction of non-vitamin K antagonist OACs [47], guideline-based screening of people ≥65 years, assuming a yield of 1.44%, is likely to be more cost-effective than some previously published estimates. However, cost-effectiveness calculations will also need to consider the possible influence of increased bleeding risk and the associated costs, including hospitalisations related to treatment with OAC for those with screen-detected AF [48].

Furthermore, it is widely acknowledged there are no published outcome data (stroke and death) for screen-detected AF [6,49]. In response to this, large screening studies with these endpoints are currently underway (e.g., ClinicalTrials.gov Identifiers NCT02743416 [STROKESTOP II] and NCT01593553). Once these and similar studies in the planning stages report, the outcome data can be combined with data from this review to calculate the number needed to treat to more precisely inform cost-effectiveness analyses and policy decisions on screening, based on the age distribution of the specific population to be screened. It appears that screening for AF in a general population is likely to be cost-effective if screening is commenced at age 65, in line with current international guidelines. However, actual cost-effectiveness will depend on the age distribution of the population to be screened as well as stroke rates in each stratum of the new AF cases discovered. Our estimates of likely yield of both AF cases and proportion of cases with an elevated calculated stroke risk enable organisations responsible for healthcare delivery to determine the best age cutoffs to suit their own budgets. For example, some organisations may decide on setting an age threshold of 70 or even 75 years, accepting a trade-off in missed opportunities to prevent strokes.

### Limitations

The heterogeneity between the included studies was high. We do not have sufficient data on the sociodemographic variables of the populations screened, or possible ascertainment biases, to explain the variance in the samples. As a logistic regression approach was chosen, we were unable to assess funnel plot asymmetry; however, the rigorous methods for identification of relevant studies will likely reduce the chance of publication bias. The detection rate of unknown AF could also be inflated in a minority of studies, as self-knowledge/recall of past AF history may be inaccurate, and studies performed in areas with reduced access to medical services may have lesser rates of previous AF diagnoses. Furthermore, the data reported in this review cannot take into account what proportion of new AF would have been detected, albeit with some delay, without screening. Few of the included studies included a control population, but in the large SAFE trial, the detection rate of new AF in practices screening people ≥65 years was 1.63% per annum, 1.04% per annum in control practices, and 1.0% in 1 year in the control group of REHEARSE-AF [26,39].

### Conclusions

People detected with new AF through screening are at elevated calculated stroke risk: above age 65, the majority are eligible for and would benefit from OAC to prevent stroke, and >70% have at least one additional stroke risk factor other than age or sex. Screening for AF in people aged ≥65 years identifies new AF in 1.44% of those screened. The detection rate was not influenced by the screening method, recruitment setting, country, or year screened. The detection rate of new AF by screening rises progressively with age, with a male predominance in all age strata. One treatable new AF will be identified for every 83 people screened in people aged ≥65 years. Our data show that the yield and stroke risk profile of new AF are sensitive to age, so the NNS-Rx is dependent on the age distribution of the population to be screened; this information is essential for precise calculations of cost-effectiveness of different age cutoffs for screening. Screening for AF in a general population is likely to be cost-effective if screening is commenced at age 65, in line with current international guidelines. However, actual cost-effectiveness will depend on the age distribution of the population to be screened, as well as stroke rates in each age stratum of the new AF cases discovered.

## Supporting information

S1 PRISMA checklist (DOC)

S1 Text Study data collected. (DOCX)

S2 Text Statistical analysis plan. (DOCX)

## Abbreviations

AF: atrial fibrillation
ECG: electrocardiogram
ESC: European Society of Cardiology
NNS: number needed to screen
NNS-Rx: NNS to identify one treatable new AF case
OAC: oral anticoagulation
SAFE: screening for atrial fibrillation in the elderly
