# Supplementary material for: Estimated stroke risk, yield, and number needed to screen for atrial fibrillation detected through single time screening: a multicountry patient-level meta-analysis of 141,220 screened individuals
Source: PLoS Med. 2019 Sep 25;16(9):e1002903. doi: 10.1371/journal.pmed.1002903 (PMC6760766; doi:10.1371/journal.pmed.1002903)
Supplement: S1 Text — (DOCX) [file pmed.1002903.s002.docx]

**S1: STUDY DATA COLLECTED**

*Study descriptive data*

- Study name
- Country
- Geographical region
- Screening setting (Community/Population, Outpatient Clinic, General Practice, Pharmacy)
- Urban or Rural population
- Screening method to detect AF (12-lead ECG, single-lead ECG, pulse palpation; modified blood pressure device)
- Screening age eligibility
- Years screening performed

*Aggregate data on number screened and identified with atrial fibrillation*

- Total number screened
- Total number of cases with new AF
  - Stratified by age groups: <60; 60-64; 65-69; 70-74; 75-79; 80-84; ≥85 years
  - Stratified by sex

*Patient level data for cases of newly identified AF*

- Age (<60; 60-64; 65-69; 70-74; 75-79; 80-84; ≥85 years)
- Sex (male; female)
- CHA_2_DS_2_-VASc score (stroke risk score ranging from 0-9)
- Oral-anticoagulation recommendation for stroke prevention (not recommended; consider treatment; Class-1 recommendation)
- Presence of stroke risk factors other than age or sex (yes; no)
