# Supplementary material for: Estimated stroke risk, yield, and number needed to screen for atrial fibrillation detected through single time screening: a multicountry patient-level meta-analysis of 141,220 screened individuals
Source: PLoS Med. 2019 Sep 25;16(9):e1002903. doi: 10.1371/journal.pmed.1002903 (PMC6760766; doi:10.1371/journal.pmed.1002903)
Supplement: S2 Text — (DOCX) [file pmed.1002903.s003.docx]

STATISTICAL ANALYSIS PLAN

**Meta-Analysis of the yield and stroke risk of atrial fibrillation detected through screening. A collaboration of 19 screening studies**

# Synopsis and aims

International guidelines recommend screening for atrial fibrillation (AF) in people aged ≥65 years, as both AF prevalence and stroke risk increase substantially from age 65. Historically, the evidence to support AF screening has been extrapolated from studies of people with clinically or incidentally diagnosed AF, as there are no large studies or databases describing screen-detected AF. The actual stroke risk profile of people with screen-detected AF is not well described in the literature. Further, the diagnostic yield of AF screening is uncertain across individual age groups, and across different geographical populations.

Therefore, this study aims to determine the optimal age for commencing screening, based on yield and stroke risk of the new cases identified. Specifically, is the recommendation for screening to start at age ≥65 years reasonable, or should a different age (e.g. age ≥70, or ≥60) be recommended.

Specifically, this study will:

1. Determine the detection rate of new AF identified through screening in age ≥65 years
2. Examine the detection rate of new AF across age subgroups
3. Examine the stroke risk (CHA_2_DS_2_-VASc score) for people with screen-detected AF across age subgroups
4. Examine eligibility for oral anticoagulation (OAC) treatment for people with screen-detected AF across age subgroups

# Study population

Atrial fibrillation screening studies from within the last 12 years, which screened a general population for atrial fibrillation using an internationally accepted method for screening

## 2.1 Inclusion criteria

Studies that:

- evaluated a general ambulant population;
- within their screened population, people over 65 years were included;
- used a valid method to identify AF, as accepted by the 2016 ESC AF guidelines;
- assessed the rate of newly identified AF using a single time-point screen;
- distinguished between newly identified AF and previously diagnosed AF;
- identified at least 10 participants with newly identified AF; and
- collected participant age for all participants screened.

## 2.2 Exclusion criteria

Studies that:

- performed repeated screening over a period to identify unknown AF
- targeted screening at a specific sub-group of a population (e.g. limited age range, hypertension, post stroke)

# Study design

## 3.1 Systematic Review

Relevant studies were identified through an electronic database search of MEDLINE, Pubmed and Google search engine. The keyword search terms used were: atrial fibrillation AND screening OR incidence OR prevalence OR detection OR identification. Additional studies were also identified through a list of studies obtained through the AF-SCREEN International Collaboration of screening groups. To ensure a relevant contemporary sample was obtained, limits were applied to the years 2007 onwards, and humans only. All languages were included.

The final search was performed in February 2018 and was not updated further due to the practicalities of time-lags of up to 5 months between sending the invitation to collaborate and receiving the final correct complete data set from each study.

## 3.2 Invitation to collaborate

Primary and/or senior study authors from all identified eligible studies were contacted via email. The email contained an explanation of the proposed study and an invitation to collaborate.

## 3.3 Data amalgamation and cleansing

Data from each study were exported into an Excel data sheet and checked three times for incorrect entries, including new AF identified that were younger than the age groups screened. Any data anomalies were amended by clarification with the primary authors from each study.

## 3.4 Data

*Study descriptive data*

- Study name
- Country
- Geographical region
- Screening setting (e.g. community, general practice, pharmacy)
- Urban vs Rural population
- Screening method to detect AF (e.g. 12-lead ECG, single-lead ECG, pulse palpation)
- Age range screened (e.g. limited to ≥65 years)
- Year screening performed

*Aggregate data on number screened and identified with atrial fibrillation*

- Supplied in age groups: <60; 60-64; 65-69; 70-74; 75-79; 80-84; ≥85 years
- Total number screened (available for all 19 studies)
- Total number of cases with new AF (available for all 19 studies)
- Total number screened (by sex) (available for 18/19 studies)
- Total number of cases with new AF (by sex) (available for all 19 studies)

*Patient level data are available for case of newly identified AF*

- Age (<60; 60-64; 65-69; 70-74; 75-79; 80-84; ≥85 years)
- Sex (male; female)
- CHA_2_DS_2_-VASc score (stroke risk score ranging from 0-9)
- Oral anticoagulation recommendation for stroke prevention (not recommended; consider treatment; Class-1 recommendation)
- Presence of stroke risk factors other than age or sex (yes; no)

# Study outcomes

## 4.1 Primary outcome

- Detection rate for cases of new AF identified through screening for **age ≥65 years** (reported as rates [cases/100 persons screened] and 95% confidence intervals)

## 4.2 Secondary outcomes

- Detection rate for cases of new AF identified through screening stratified according to each age group (<60; 60-64; 65-69; 70-74; 75-79; 80-84; ≥85 years) (reported as rates [cases/100 persons screened] and 95% confidence intervals)
- CHA_2_DS_2_-VASc stroke risk score; stratified according to age group (reported as means and 95% confidence intervals)
- Guideline-based eligibility for oral anticoagulation of newly identified AF; stratified according to age group (reported as number and percentage)
- The number of additional stroke risk factors other than age and sex; stratified according to age group (reported as number and percentage)
- Number needed to screen to identify 1 new AF case for age ≥65 years; and stratified across age subgroups
- Number needed to screen to identify 1 treatable new AF case (i.e. new AF with a Class-1 recommendation to prescribe OAC) for age ≥65 years; and stratified across age subgroups

# Statistical analysis

## 5.1 Descriptive analysis

Descriptive analyses will be carried out for

- Characteristics of participating studies (see example table below)
- Total numbers of: AF identified through screening according to sex, and total numbers screened (see example table below)

## 5.2 Detection rate of new AF (aggregated data)

Data from all studies will be imported into SAS GLIMMIX. ‘Detection rate of new AF’ is defined as a proportion, with a binary outcome of either AF detected, or no AF detected. Therefore, this analysis assumes that the data follows a binomial distribution. Confidence intervals for each study will be calculated using an exact binomial approach.

Statistical heterogeneity between studies will be assessed using the study-level random effect and standard error.

The pooled event rate and variance will be calculated using a random-effects logistic regression with study included as a random effect and covariates of geographical region; urban/rural population; screening method/device; screening setting/design; year study performed; age range targeted; age; and sex. The quality of the statistical model will be assessed using Akaike’s Information Criterion. Results will be reported for age ≥65 years (i.e. 65-69; 70-74; 75-79; 80-84; ≥85 years grouped together); and also stratified according to each age group (<60; 60-64; 65-69; 70-74; 75-79; 80-84; ≥85 years).

As a consequence of choosing this model of analysis, traditional assessment of publication bias cannot be performed.

Sensitivity analysis will be performed to assess the influence of each study, by removing one single study each time and repeating the analysis.

## 5.3 Stroke risk profile of new AF cases (patient level data)


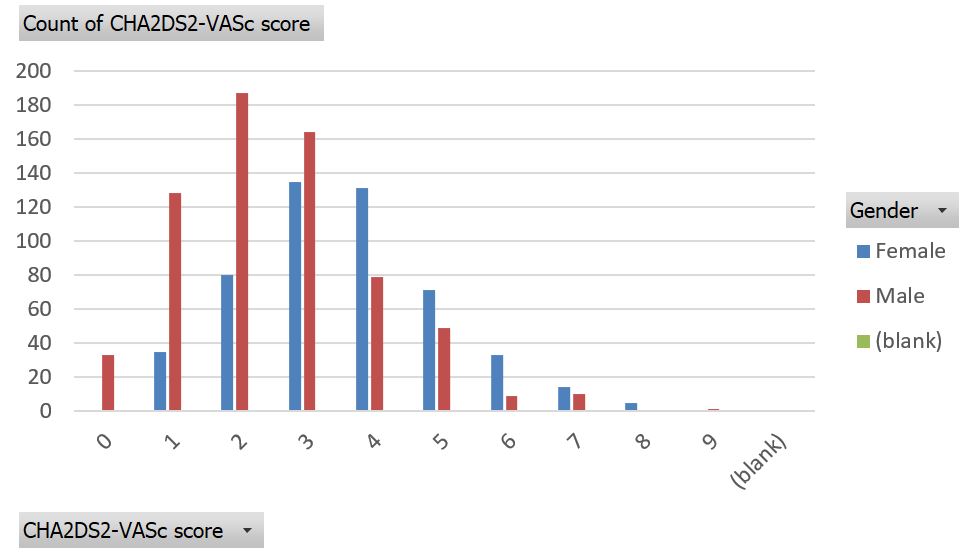
CHA_2_DS_2_-VASc score data are ‘count data’. As the CHA_2_DS_2_-VASc score data has a Poisson distribution, random-effects Poisson regression modelling will be performed for CHA_2_DS_2_-VASc score; using study as a random effect and fixed effects of sex, age group, and geographical region. The quality of the statistical model will be assessed using Akaike’s Information Criterion.

CHA_2_DS_2_-VASc scores will be reported as means and 95% confidence intervals, stratified according to age group (<60; 60-64; 65-69; 70-74; 75-79; 80-84; ≥85 years).

Guideline recommendation for oral-anticoagulation will be calculated for the pooled data according to the following formula, and reported as pooled number and percentages for each category; stratified according to age group (<60; 60-64; 65-69; 70-74; 75-79; 80-84; ≥85 years)

- Not recommended: CHA_2_DS_2_-VASc score: men=0; women=1
- Consider oral anticoagulation: CHA_2_DS_2_-VASc score: men=1; women=2
- Class-1 recommendation: CHA_2_DS_2_-VASc score: men≥2; women≥3

The number of additional stroke risk factors other than age and sex will be calculated for each person with new AF using the formula (CHA_2_DS_2_-VASc score – female sex point - age points); and reported as a pooled percentage of all new AF; stratified according to age group (<60; 60-64; 65-69; 70-74; 75-79; 80-84; ≥85 years).

## 5.4 Number needed to screen

Number needed to screen to identify one new AF case will be calculated using the inverse of the determined yield (i.e. number screened/number with newly-identified AF); stratified according to age group (<60; 60-64; 65-69; 70-74; 75-79; 80-84; ≥85 years).

Number needed to screen to identify ‘one treatable AF’ will be calculated using the inverse of the determined yield (i.e. number screened/number with newly-identified AF with a Class-1 recommendation for oral anticoagulation); stratified according to age group (<60; 60-64; 65-69; 70-74; 75-79; 80-84; ≥85 years).
